# Single-cell and Spatial Transcriptomics Reveals Ferroptosis as The Most Enriched Programmed Cell Death Process in Hemorrhage Stroke-induced Oligodendrocyte-mediated White Matter Injury

**DOI:** 10.7150/ijbs.96262

**Published:** 2024-07-08

**Authors:** Lingui Gu, Hualin Chen, Ruxu Geng, Mingjiang Sun, Qinglei Shi, Yihao Chen, Jianbo Chang, Junji Wei, Wenbin Ma, Jiashun Xiao, Xinjie Bao, Renzhi Wang

**Affiliations:** 1 Peking Union Medical College Hospital, Chinese Academy of Medical Sciences and Peking Union Medical College, Beijing 100730, China.; 2 Eight-year Medical Doctor Program, Chinese Academy of Medical Sciences and Peking Union Medical College, Beijing 100730, China.; 3 Beijing Neurosurgical Institute, Beijing Tiantan Hospital, Capital Medical University. Beijing, 100070, China.; 4 Research Institute of Big Data, Chinese University of Hong Kong (Shenzhen) School of Medicine, Shenzhen, China.; 5 State Key Laboratory of Common Mechanism Research for Major Diseases, Beijing, China.; 6 School of Medicine, The Chinese University of Hong Kong, Shenzhen, Guangdong, 518172, China.

**Keywords:** single-cell and spatial transcriptome, intracerebral hemorrhage, microglia, oligodendrocytes, ferroptosis

## Abstract

Intracerebral hemorrhage (ICH) is a severe stroke subtype with limited therapeutic options. Programmed cell death (PCD) is crucial for immunological balance, and includes necroptosis, pyroptosis, apoptosis, ferroptosis, and necrosis. However, the distinctions between these programmed cell death modalities after ICH remain to be further investigated. We used single-cell transcriptome (single-cell RNA sequencing) and spatial transcriptome (spatial RNA sequencing) techniques to investigate PCD-related gene expression trends in the rat brain following hemorrhagic stroke. Ferroptosis was the main PCD process after ICH, and primarily affected mature oligodendrocytes. Its onset occurred as early as 1 hour post-ICH, peaking at 24 hours post-ICH. Additionally, ferroptosis-related genes were distributed in the hippocampus and choroid plexus. We also elucidated a specific interaction between lipocalin-2 (LCN2)-positive microglia and oligodendrocytes that was mediated by the colony stimulating factor 1 (CSF1)/CSF1 receptor pathway, leading to ferroptosis induction in oligodendrocytes and subsequent neurological deficits. In conclusion, our study highlights ferroptosis as the primary PCD mechanism, emerging as early as 1 hour post-ICH. Early therapeutic intervention via the suppression of microglial LCN2 expression may alleviate ferroptosis-induced damage in oligodendrocytes and associated neurological deficits, thus offering a promising neuroprotective strategy following ICH.

## Introduction

Intracerebral hemorrhage (ICH) is a cerebrovascular disease with high morbidity, mortality, and disability^1^. Despite advances in surgical techniques, such as conventional craniotomy and minimally invasive approaches (including stereotactic and endoscopic surgery), the prognosis for patients has remained largely unchanged^2^, ^3^.

Oligodendrocytes are crucial in the central nervous system, where they facilitate myelination. In a process known as oligodendrogenesis, oligodendrocyte precursor cells (OPCs) mature into myelin-producing cells, which are essential for white matter repair because of their role in axonal ensheathment and neuronal metabolic support^4^. Hemorrhagic stroke-induced oligodendrocyte loss leads to axonal damage and subsequent neurological deficits^5^. Activated oligodendrocytes have therapeutic potential for remyelination, thus mitigating white matter injury post-ICH. Notably, strategies for preventing oligodendrocyte death and promoting myelin restoration are fundamental for neurological recovery^6^. Post-ICH, blood-brain barrier disruption, neuroinflammation, oxidative stress, and neurotoxic effects trigger various forms of programmed cell death (PCD)^7^, such as apoptosis, pyroptosis, ferroptosis, autophagy, and necrosis, thus influencing post-ICH immune responses^8^. Ferroptosis is characterized by iron-dependent oxidative injury, and is increasingly recognized in conditions including hemorrhagic stroke, Alzheimer's disease, and Parkinson's disease, which indicates possible novel therapeutic approaches for these disorders^9^. Pre-clinical studies have demonstrated the reparative effect of PCD inhibition on nerve damage subsequent to ICH^10^. For example, in pre-clinical studies, PCD inhibition reportedly has reparative effects post-ICH, although the optimal strategy remains unclear. The targeting of oligodendrocyte preservation and remyelination may thus offer a novel therapeutic avenue, and personalized medicine might lead to optimized outcomes by tailoring treatments to individual cases.

In the present study, we first used single-cell and spatial transcriptomic techniques to explore the different PCD processes that occur after different stages of ICH, and then used multiple algorithms to calculate and experimentally validate the results. Importantly, ferroptosis emerged as the primary PCD mechanism, notably affecting mature oligodendrocytes following ICH. Its onset was evident within just 1 hour post-ICH, and peaked at 24 hours post-ICH. Moreover, our investigation revealed a specific interplay between lipocalin-2-positive (LCN2^+^) microglia and oligodendrocytes, which was orchestrated through the colony stimulating factor 1 (CSF1)/CSF1 receptor (CSF1R) pathway. This intricate interaction ultimately triggered ferroptosis induction in oligodendrocytes, leading to the subsequent onset of neurological deficits. Together, our findings suggest that microglia with increased LCN2 expression may instigate ferroptosis in oligodendrocytes through the CSF1/CSF1R signaling pathway, thus exacerbating neurological deficits within the hippocampus and choroid plexus subsequent to ICH.

## Methods and materials

### Animals

All animal experiments were conducted using male Sprague Dawley rats (n = 90; weight = approximately 250 g) that were sourced and bred at the Animal Center of Spiff Biotechnology Co., Ltd. (Beijing, China). All rats were maintained at room temperature (22°C ± 1°C) with a 12-hour day/night cycle (humidity: 60% ± 5%) and unrestricted access to food and water. The experimental protocols were reviewed and approved by the Animal Ethics Committee of the Chinese Academy of Medical Sciences and Peking Union Medical College (Approval No. XHDW-2022-085). Our animal studies adhered to the guidelines provided by the National Institutes of Health for the care and use of laboratory animals, as well as the ARRIVE (Animal Research: Reporting In Vivo Experiments) guidelines.

### ICH model induction

The ICH model was created by injecting autologous blood into the brain. Each rat was intubated and kept under anesthesia using 3% isoflurane mixed with oxygen at a ratio of 7:3. This was performed using a rodent ventilator from Harvard Apparatus (Holliston, MA, USA). Each rat received 100 μL of autologous arterial blood, which was taken from the central artery of the tail. The blood was injected into the basal ganglia (0.26 mm anterior, 3.0 mm left lateral, and 6.0 mm deep) using stereotaxic techniques, without the use of anticoagulants. To avoid any backflow, the needle was kept in position for at least 10 min after the injection. After removing the needle, the burr hole was packed with bone wax, the skin was sutured, and the region was sterilized. Rat body temperature was constantly regulated at 37°C using an electric blanket. Following the surgical procedure, the effectiveness of the ICH model was assessed using the Bederson score. Models that were considered ineffective, such as rats who were asymptomatic or died before being sacrificed, were excluded from the analysis.

### Intracerebroventricular injection of short interfering RNA (siRNA) or scrambled siRNA

The following two types of siRNAs were used: (1) disorganizing rat LCN2 mRNA (si-LCN2), to silence its transcription; and (2) scrambled siRNA (si-NC) (both from Ribobio). According to the manufacturer's instructions for Entranster-in vivo RNA transfection reagent (Engreen, Shanghai, China), 500 pmol si-LCN2 and 500 pmol si-NC were dissolved in 5 μL RNase-free water. Next, 10 μL Entranster-in vivo RNA transfection reagents were added to 5 μL si-LCN2 or si-NC. After mixing for 15 min, the Entranster-in vivo-siRNA mixture was intracerebroventricularly injected 48 hours prior to the induction of ICH. The two target sequences for the siRNA design were as follows: si-NC, sense (5′-3′): UUC UCC GAA CGU GUC ACG UTT, antisense (5′-3′): ACG UGA CAC GUU CGG AGA ATT; and si-LCN2, sense (5′-3′): GGU CCA GAA AGA AAG ACA ATT, antisense (5′-3′): UUG UCU UUC UUU CUG GAC CTT.

### Cell culture and preparations

The human microglial cell line HMC3 was obtained from the American Type Culture Collection (ATCC). Briefly, HMC3 was maintained in Eagle's minimum essential medium supplemented with 10% fetal bovine serum (FBS), 50 U/mL penicillin, and 50 μg/mL streptomycin in a humidified incubator. The human oligodendrocytic cell line MO3.13 was purchased from Cedarlane (Burlington, ON, Canada). Cells were cultured according to the manufacturer's protocol in Dulbecco's Modified Eagle Medium (D5796, Sigma-Aldrich, St. Louis, MO, USA) supplemented with 10% FBS (Thermo Fisher Scientific, Waltham, MA, USA), 100 μg/mL streptomycin, and 100 U/mL penicillin (both from Sigma-Aldrich) in 5% CO_2_ at 37°C. The medium was changed every 2-3 days, and cells were passaged when confluent. To induce differentiation, MO3.13 cells were incubated in the presence of 100 nM phorbol-12-myristate-13-acetate (Sigma-Aldrich) or 200 nM of the Pol I inhibitor CX-5461 (Cayman Chemical, Ann Arbor, MI, USA) in Dulbecco's Modified Eagle Medium supplemented with 1% FBS. The medium was exchanged every second day and cell morphology was analyzed daily. Cell morphology was examined using an Axiovert 40C microscope (Carl Zeiss, Jena, Germany) equipped with an A-Plan 10×/0.25 Ph1-objective^11^.

### Single-cell RNA sequencing (scRNA-seq) protocol

#### Tissue collection

Brain tissue samples were harvested from the perihematomal region (surrounding the experimentally induced lateral hematoma) of a rodent model of ICH.

#### Tissue dissociation

The tissue was first cut into small pieces, each approximately 1 mm³ in size. These pieces were then placed into a petri dish containing an appropriate volume of phosphate-buffered saline (PBS). Subsequently, the tissue pieces were transferred to a centrifuge tube; an appropriate amount of enzyme was then added, and the mixture was agitated at a specific temperature for a predetermined duration. After standing for 2-3 min, the supernatant was removed and filtered through a membrane to remove large clumps. Following centrifugation, the supernatant was discarded and the cells were resuspended in red blood cell lysis buffer, incubated for 2-3 min at room temperature, and centrifuged (120 × *g*, 4°C, 3 min). Finally, the samples were resuspended in PBS.

#### 10× Genomics scRNA-seq cell capture and cDNA synthesis

Cell suspensions (300-600 living cells/µL, determined using Count Star) were loaded onto a Chromium Single Cell Controller (10× Genomics) to generate single-cell gel beads in emulsion (GEMs) using a Single Cell 3′ Library and Gel Bead Kit 2 (10× Genomics, 120237) and Chromium Single Cell A Chip Kit (10× Genomics, 120236) according to the manufacturer's protocols. Briefly, single cells were suspended in 0.04% bovine serum albumin in PBS, and cells were added to each channel. The captured cells were lysed, and the released RNA was barcoded through reverse transcription in individual GEMs. GEMs were reverse transcribed using a C1000 Touch Therma Cycler (Bio Rad) programmed at 53°C for 45 min and 85°C for 5 min, and were held at 4°C. After reverse transcription, single-cell droplets were broken, and the single-strand cDNA was isolated and cleaned with a Cleanup Mix containing Dynabeads (Thermo Fisher Scientific). The cDNA was then generated and amplified, and its quality was assessed using an Agilent 4200 system.

### scRNA-seq library preparation

The scRNA-seq libraries were prepared using a Single Cell 3′ Library Gel Bead Kit V2 according to the manufacturer's instructions. Sequencing was performed using an Illumina Novaseq6000 with a sequencing depth of at least 100,000 reads per cell and a pair-end 120-bp reading strategy.

### Spatial transcriptome protocol

#### Staining and imaging

Cryosections were cut at 10 μm thickness and mounted onto the GEX arrays. Sections were then placed on a Thermocycler Adaptor with the active surface facing up, incubated for 1 min at 37°C, fixed for 30 min with methyl alcohol at -20°C, and stained with hematoxylin and eosin (H&E; eosin, Dako CS701; hematoxylin, Dako S3309; bluing buffer CS702). Brightfield images were taken using a Leica DMI8 whole-slide scanner at 10× resolution.

#### Gene expression and transfer

Visium spatial gene expression was processed using a Visium Spatial Gene Expression Slide and Reagent Kits (10× Genomics, PN-1000184). For each well, a slide cassette was used to create leakproof wells for adding reagents. First, 70 μL permeabilization enzyme was added and incubated at 37°C. Each well was then washed with 100 μL SSC, and 75 μL Reverse Transcription Master Mix was added for cDNA synthesis.

#### cDNA library preparation for sequencing

At the end of first-strand synthesis, the Reverse Transcription Master Mix was removed from the wells. Next, 75 μL of 0.08 M KOH was added and incubated for 5 min at room temperature. After removing the KOH, the wells were washed with 100 μL EB buffer, and 75 μL Second Strand Mix was added to each well for second-strand synthesis. The cDNA amplification was performed using a S1000TM Touch Thermal Cycler (Bio Rad). According to the manufacturer's instructions, the Visium spatial libraries were constructed using a Visium Spatial Library Construction Kit (10× Genomics, PN-1000184). The libraries were sequenced using an Illumina Novaseq6000 sequencer, with a sequencing depth of at least 100,000 reads per spot with a pair-end 120-bp reading strategy (performed by CapitalBio Technology, Beijing, China).

### scRNA-seq bioinformatic analysis

#### Seurat pipeline

Seurat (v4.3.0) workflow^12^, an R package (R v4.2.1), facilitated the downstream analysis of the Cell Ranger outputs. We aggregated data across the hyperacute (1 hour, ICH-1h), acute (24 hours, ICH-24h), and subacute (7 days, ICH-7d) phases, encompassing a primary dataset of 67,554 cells with a median gene count of 2,321 genes per cell. The initial phase involved the exclusion of low-quality cells—specifically those with anomalous unique gene counts and/or a disproportionately high mitochondrial genome read percentage. After quality control, the dataset comprised 63,211 cells, including 18,363 ICH-1h cells, 18,964 ICH-24h cells, and 25,884 ICH-7d cells. The normalization of feature expression was conducted using the *NormalizeData()* function; each cell's total expression was scaled to a factor of 10,000, followed by log-transformation. Prior to dimension reduction, the *ScaleData()* function was used for data scaling. Feature selection was facilitated using the *FindVariableGenes()* function, which identified the 2,000 genes with the most variation across cells. These variable features underpinned the principal component analysis using the *RunPCA()* function, which focused on the top 30 principal components for cell clustering. Clustering was executed via the *FindClusters()* function using the Louvain algorithm, and resulted in 33 distinct clusters. Cluster-specific markers were discerned by contrasting gene expression within clusters against other cells using the *FindMarkers()* function. Data visualization, post-filtering, normalization, and scaling were achieved using the Uniform Manifold Approximation and Projection (UMAP) algorithm integrated within the Seurat workflow. All plots were generated using the built-in functions of Seurat, Scanpy (v1.9.6)^13^ workflows, and the ggplot2 (v3.2.1) package.

#### scRNA-seq enrichment analyses

Gene sets related to PCD, including apoptosis, autophagy, ferroptosis, necroptosis, and pyroptosis, were procured from a previous study by Liu et al^14^. The homologene (v1.4.68) R package was used to translate human genes into their rat homologs. The *AddModuleScore()* function in Seurat, *AUCell_calcAUC()* function in AUCell (v1.20.1), and *AddModuleScore_UCell()* function in UCell (v2.2.0) were used to quantify the module scores and the fraction of enrichment for PCD-related gene expression in single cells. irGSEA (v2.1.5), an R package, was used to determine the enrichment of one specific gene set in a certain cell type, and was based on the built-in single-cell rank-based gene set enrichment analysis, which included the following nine well-known methods: GSEA, GSVA, PLAGE, Zscore, AddModuleScore, ssGSEA, AUCell, UCell, and singscore. In brief, we calculated the differentially expressed gene sets for each cell subpopulation within the gene set enrichment score matrices using the Wilcoxon test. Subsequently, we performed a comprehensive evaluation of the differential analysis results using the rank aggregation algorithm (robust rank aggregation) from the RobustRankAggreg package. This approach was used to identify gene sets that were significantly enriched across most gene set enrichment analysis methods.

#### Trajectory analysis

RNA velocity assessment was performed using the python packages velocyto (v0.17)^15^ and scVelo (v0.2.3)^16^. Specifically, the 10× velocyto pipeline was used to process filtered Cell Ranger-generated BAM files to quantify the spliced and unspliced reads per sample. To infer single-cell RNA velocity, each cell's latent time was estimated using the scVelo dynamical model. Subsequently, the R package Monocle (version 2.26.0)^17^ was used to explore the single-cell pseudotime trajectory.

#### Oligodendrocyte lineage differentiation matching

We used a published dataset of oligodendrocyte lineage cells (GSE75330)^18^ in which Marques et al. defined five stages of oligodendrocyte lineage differentiation. To match the current scRNA-seq data to these oligodendrocyte lineage stages, we calculated the Pearson's correlation coefficients of the gene expression profiles between the cells from our seven oligodendrocyte subpopulations and cells from these stages.

#### Cell-cell interaction analysis

The nichenetr (v2.0.0)^19^/multinichenetr (v1.0.1)^20^ R package was used for the computational exploration of intercellular communication. Fundamentally, NicheNet methodology leverages gene expression data from human or mouse cells in interaction, and integrates these data with a pre-existing model that synthesizes known ligand-to-target signaling pathways. This framework enables the prediction of ligand-receptor interactions that may influence gene expression modifications in cells of interest. MultiNicheNet, an advancement of NicheNet, has been specifically engineered for the differential analysis of cell-cell communication in single-cell transcriptomics, and can accommodate complex multi-sample and multi-condition designs. The overarching aim of the use of this analytical suite was to elucidate variations in intercellular communication across distinct sample groups.

#### Gene Ontology Biological Process (GO_BP) enrichment analyses

Enrichment analysis of GO_BP terms in OLs and OPCs was performed using the upregulated genes specific to each oligodendrocyte subset. This was facilitated by the Single-Cell Pipeline (https://zhanghao-njmu.github.io/SCP/), a comprehensive tool that was designed for detailed single-cell data analysis.

### Spatial transcriptomic analyses

#### SpatialScope pipeline

The SpatialScope (v1.0.0) workflow^21^ is a python package for integrating spatial and single-cell transcriptomic data by leveraging deep generative models. SpatialScope is designed to resolve spot-level seq-based spatial transcriptome data (e.g., Visium) into single-cell resolution. The workflow of SpatialScope consists of three ladder-like steps. In step 1, the number of cells within each spot in Visium data is quantified using the paired H&E-stained histological images. In step 2, the cell-type labels for individual cells within the spot are identified using scRNA-seq data as a reference. In step 3 (which is optional), by conditioning the inferred cell type labels, gene expression decomposition is performed to transform the spot-level gene expression profile into single-cell resolution. In the present study, because of the limitation of revision time, we only applied the first two steps of SpatialScope to our Visium data. In step 1, we followed the online guidelines and used StarDist to perform nuclear segmentation in the high-resolution H&E-stained histological images. The parameter *--prob_thresh* of the *Nuclei_Segmentation.py* script was set as low as 0.1, to capture as many cells as possible. After segmentation, we observed that the numbers of segmented nuclei ranged from 1 to 19 across Visium spots, with a median of three cells. In step 2, we used the *Cell_Type_Identification.py* script to infer the cell-type labels for the segmented cells within the spot; we set the parameter *--nu* as 1 to incorporate modest spatial smoothness. For the Visium data from the ICH-24h group, we leveraged the corresponding scRNA-seq data of the ICH-24h group as a reference to transfer cell-type annotations. This allowed us to align the spatial transcriptomics with the scRNA-seq-based annotations, thus providing a comprehensive understanding of the cellular composition within the tissue samples. For Visium data from the ICH-1h group, the same strategy was applied.

#### Spatial mapping of cell states using cell2location

Cell2location (v0.1.3)^22^, a python package, was used to decipher and spatially position single-cell clusters within the spatial transcriptomic spots. Initially, we derived reference signatures for the cellular states from scRNA-seq data for each area, applying a negative binomial regression model. The regression model was tailored for single-cell data and commenced with standard configurations, running through 5,000 epochs. This model subsequently underwent training, capped at 30,000 epochs. The deduced reference signatures of cell types facilitated the cell2location cell-type mapping in pertinent regions, thus enabling an estimation of the prevalence of each cell state in every spot. To assess the reliability of cell2location, we used CellTrek^23^ software for the combined embedding of scRNA-seq data on a spatial scale, which enabled us to examine the co-localization tendencies among diverse cellular populations.

### Immunofluorescence staining

The animals were anesthetized and transcranially perfused with 200 mL ice-cold PBS and 200 mL 4% paraformaldehyde 24 hours post-ICH. The brain was removed and fixed overnight in 4% paraformaldehyde before being cryoprotected in 20% and then 30% sucrose (for 1 day each). Next, each brain was embedded in optimal cutting temperature compound, frozen at -80°C, and cut into 8-μm coronal slices using a CM1860 cryostat (Leica Microsystems, Germany). The brain slices were then incubated overnight at 4°C with anti-oligodendrocyte transcription factor 2 (OLIG2; 1:100, Abcam) and anti-glutathione peroxidase 4 (GPX4; 1:100, Cell Signaling Technology) primary antibodies for double immunohistochemical labeling. After three washes, the sections were incubated with suitable secondary antibodies (1:200, Bioss, China) for 1 hour at 37°C. A fluorescent microscope (U-HGLGPS, Olympus, Japan) was then used to take pictures from at least three randomly chosen fields per section, and from two randomly selected sections from the main hemorrhagic region, for each rat. For microphotograph analysis, cellSens Standard was used. Cells were seeded on glass coverslips in six-well plates. The cells were fixed in 4% paraformaldehyde at room temperature for 20 min after different treatments; this was followed by three washes in PBS with Tween 20. Following 15 min of permeabilization with 0.1% Triton X-100 in PBS, the cells were washed, blocked with 5% bovine serum albumin in PBS for 1 hour, and incubated overnight with anti-APC (Abcam) and anti-GPX4 antibodies at 4°C. After washing with PBS, the cells were treated with fluorescein isothiocyanate-conjugated secondary antibodies (1:1,000) at room temperature for 1 hour. The cells were then washed with PBS, mounted in Fluoroshield with 4',6-diamidino-2-phenylindole, and imaged using a confocal microscope.

### Western blot

Proteins were electrophoresed in 10% sodium dodecyl sulfate-polyacrylamide gels and transferred to polyvinylidene fluoride membranes using standard electroblotting procedures. The membranes were then blocked for 1 hour at 37°C followed by incubation with the following primary antibodies overnight at 4°C: anti-acyl-CoA synthetase long-chain family member 4 (ACSL4; 1:1,000, Abcam), anti-cystine/glutamate transporter (xCT; 1:1000, Abcam), anti-ferritin heavy chain (FTH1; 1:1,000, Abcam), anti-GPX4 (1:1,000, Cell Signaling Technology), anti-LCN2 (1:1000, Abcam), and anti-β-actin (1:5,000, Proteintech, China). The secondary antibodies (ZSGB-BIO) were incubated for 1 hour at 37°C. Next, the protein bands were visualized using enhanced chemiluminescence, and relative protein quantities were determined using ImageJ software (National Institutes of Health, Bethesda, MD, USA).

### Measurement of intracellular reactive oxygen species (ROS)

The 2′,7′-dichlorodihydrofluorescein diacetate (DCFH-DA) test was used to detect intracellular ROS. In this test, DCFH is oxidized to fluorescent DCF by ROS. In 96-well plates, neutrophils were rinsed in PBS and treated with 10 μM DCFH-DA at 37°C for 30 min in the dark. A fluorescent microplate reader (Spectra Max Gemini EM, Molecular Devices, Sunnyvale, CA, USA) was then used to detect 485- and 535-nm fluorescence. Cells were imaged using a laser-scanning confocal microscope (FV1000, Olympus) and FV10-ASW 4.0 VIEW by investigators who were blinded to treatments.

### Transmission electron microscopy

Differently treated cells were fixed overnight at 4°C in 2.5% glutaraldehyde, washed twice with 0.1 M phosphate buffer for 15 min, and post-fixed with 2-3 drops of 1% osmic acid at room temperature for 1.5 hours. The cells were then dehydrated through 30%, 50%, 70%, 80%, 95%, and 100% ethanol for 15 min each, or were left at 4℃ overnight in 70% ethanol and then in 100% ethanol. The ethanol was replaced with 100% acetone twice, for 15 min each time. Next, the cells were incubated in 1:1 Epon 812/acetone for 2-3 hours before being left in pure Epon 812 overnight. The samples were then polymerized in fresh Epon 812 at 60℃ for 48 hours before being roughly trimmed to expose the sample and smooth the area. A diamond knife was used to cut 70 nm sections, which were scanned using a Hitachi H7500 Transmission Electron Microscope.

### Neurobehavioral functional tests

Neurobehavioral functions were assessed by a blinded investigator using the modified Garcia test, rotarod test, and Morris water maze at 24 and 72 hours following ICH, as previously described^24^, ^25^. In the modified Garcia test, seven distinct aspects were evaluated, including spontaneous activity, axial sensation, vibrissae touch, limb symmetry, lateral turning, forelimb walking, and climbing. Each test was scored from either 0-3 or 1-3 to yield a maximum deficit score of 21, with a higher score indicating superior neurological function. The rotarod test was used to evaluate overall motor coordination and motor learning in rats. The rats underwent three trials per day (with a 1-hour intertrial interval) over a span of 3 consecutive days. The rats were placed on a rotarod apparatus (Ugo Basile, Varese, Italy) that started at 5 rotations per min (rpm). The speed of the rotarod was gradually increased from 5-40 rpm over a 5-min duration. The time taken for the rat to fall from the rotating beam was recorded for each animal. However, a trial was terminated when the rat clung to the beam without walking for three consecutive turns, or when a maximum of 300 s had elapsed. Spatial learning and memory were assessed using the Morris water maze. This involved a blue circular pool filled with opaque water, divided into four virtual quadrants (northwest, southwest, southeast, and northeast). The experimental protocol consisted of 4 training days followed by a probe trial day. During the training days, the rats learned the location of a hidden platform within the pool, using visual cues around the maze. Each rat performed four trials per day, with the starting quadrant varying in a clockwise direction for each trial. Performance metrics included escape latency, swimming path length to the platform, and swimming speed, all of which were measured and analyzed using Ethovision XT10 software (Noldus, Wageningen, Netherlands).

### Statistical analysis

Data were analyzed using GraphPad Prism (version 9.0, GraphPad Software, Inc.) and Image J (version 1.8.0) using one-way analysis of variance and the Student-Newman-Keuls test. Results are presented as the mean ± standard deviation. A P-value <0.05 indicates significance.

## Results

### Single-cell and spatial transcriptome analyses of rat brain glia following ICH

**Figure 1:**
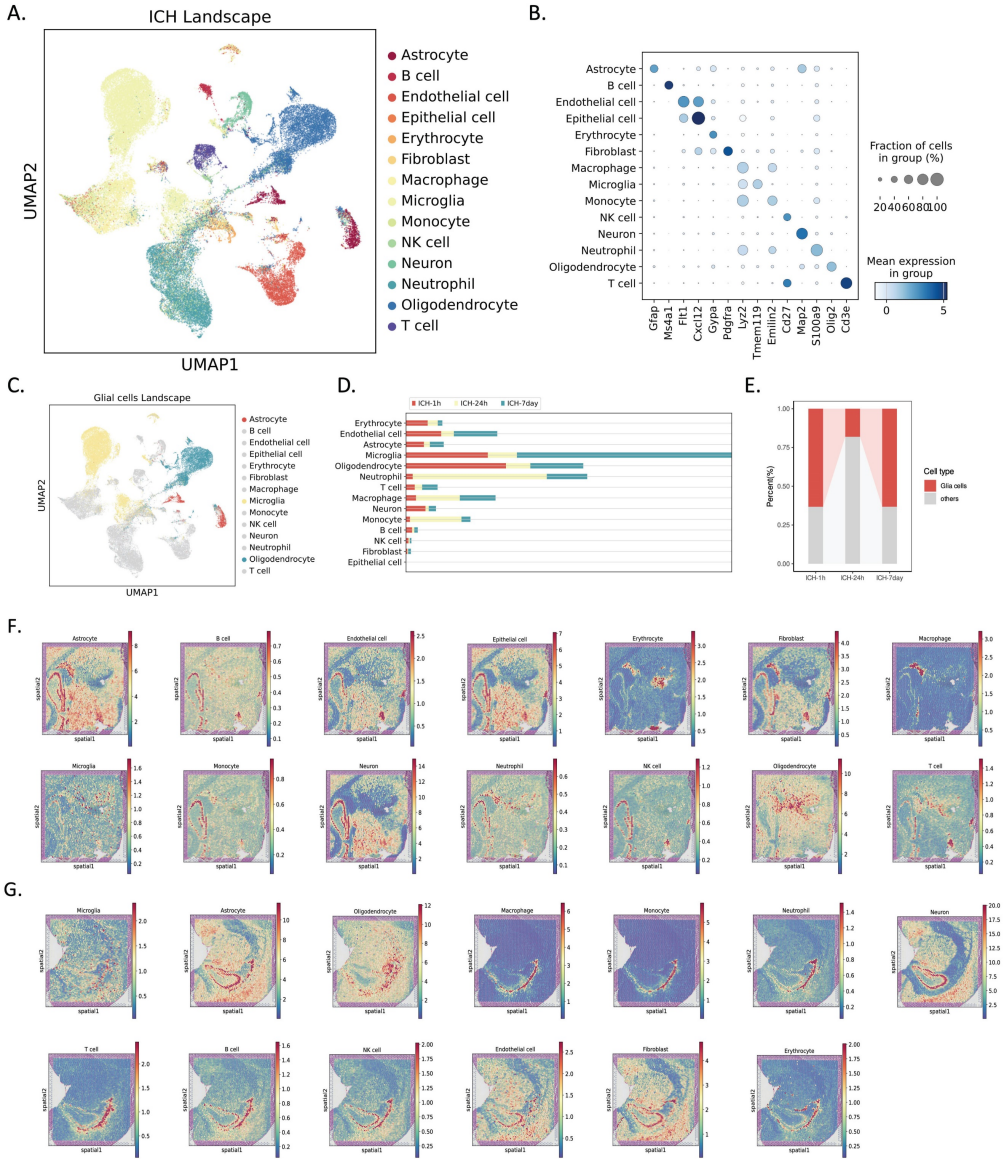
** Single-cell and spatial transcriptomic profiling of rat brain tissues in the hyperacute (1 hour, ICH-1h), acute (24 hours, ICH-24h), and subacute (7 days, ICH-7d) phases after hemorrhagic stroke.** (A) UMAP of 63,211 single-cell transcriptomes (ICH-1h: n = 18,363, ICH-24h: n = 18,964, ICH-7d: n = 25,884; three rats per group). Cell clusters were color coded and annotated post hoc based on their transcriptional profile identities. (B) Dot plot showing the distribution of expression levels of well-known representative cell-type-enriched marker genes across all 14 cell types (n = 63,211 cells). (C) UMAP of 31,437 glial cells (astrocytes: n = 2,193, microglia: n = 18,938, oligodendrocytes: n = 10,306) from the single-cell landscape. Glial cell clusters are color coded corresponding to (A), and the left cell clusters are decolored. (D) Bar plot demonstrating the cell numbers of each major cluster at different time points. Time points are color coded. The x-axis indicates the cell number. (E) Bar plot demonstrating the percentage of glial cells across three time points. Glial cells are red coded. The y-axis indicates the relative percentage. (F, G) Overlay of H&E staining of tissue sections and spatial transcriptome spots of each major cell type, predicted by cell2location, in the ICH-1h (top) and ICH-24h (bottom) rat brain.

To study dynamic changes in the transcriptomes of individual cells at different stages (hyperacute, acute, and subacute ICH), we performed scRNA-seq on nine brain hemispheres from young adult rats (8-10 weeks old) at three different times: 1 hour after ICH induced by autologous blood stereotaxic injection, 24 hours after ICH, and 7 days after ICH (n = 3 rats per group). We next annotated the cells using SingleR and CellMarker, and identified 14 cell types using core markers (Figure 1A, B). Of these, we examined glial cells (Figure 1C). Cell proportions were assessed at various times (Figure 1D, E). Hierarchical clustering of cells based on cell composition in their spatial neighborhood generated spatial clusters that naturally segmented the imaging region at 1 and 24 hours post-ICH. The H&E staining of tissue slices and spatial transcriptome spots of each main cell type were predicted by cell2location (Figure 1F, G).

### Analysis of PCD processes identified ferroptosis as the predominant PCD type in oligodendrocytes following ICH

**Figure 2:**
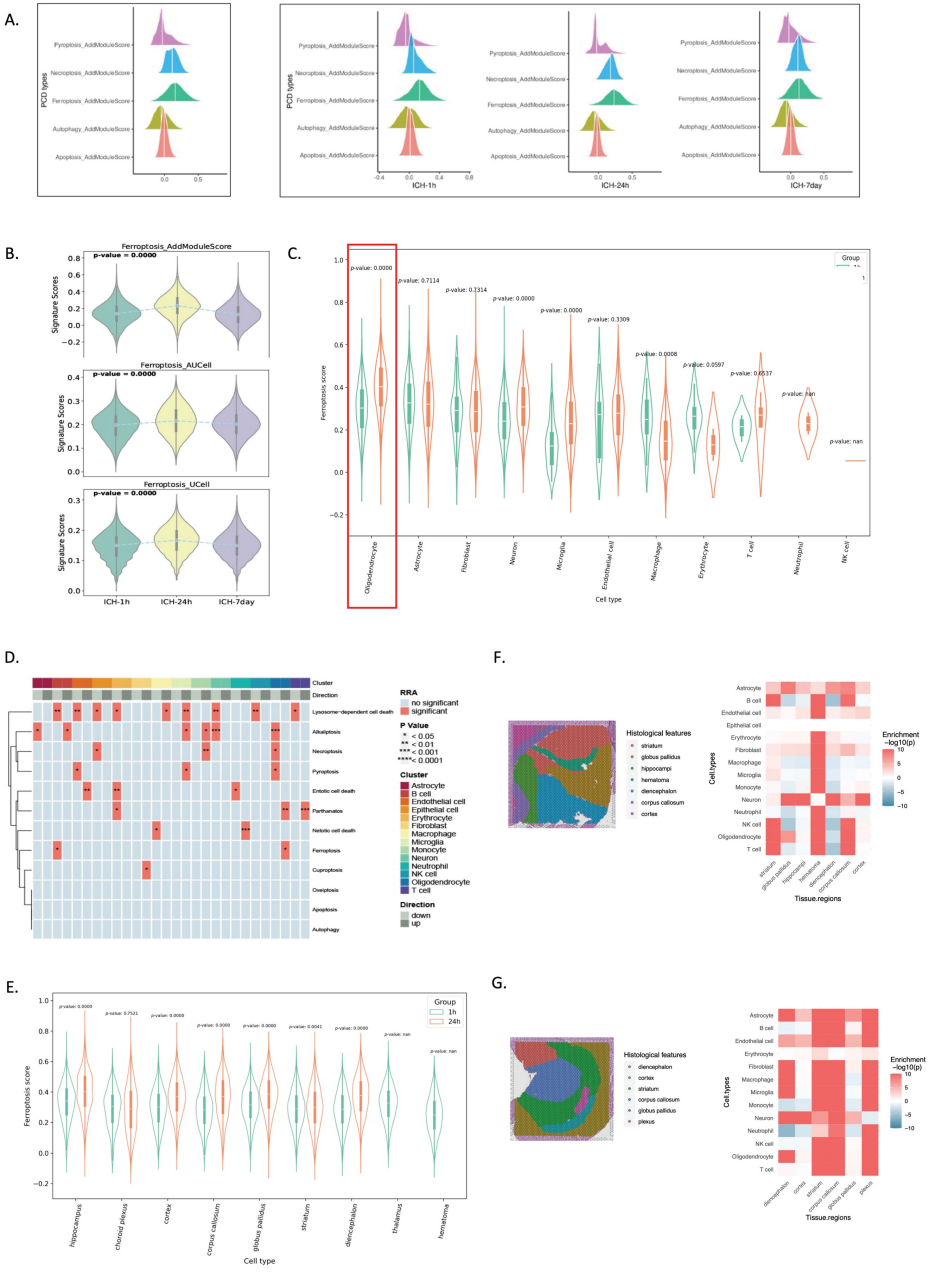
** scRNA-seq and spatial RNA-seq analyses reveal that ferroptosis preferably occurs in oligodendrocytes at 24 hours post-ICH.** (A) Illustration of five different PCD scores determined using the *AddModuleScore()* function in Seurat across three time points. (B) Violin plots illustrating the signature scores of ferroptosis across three time points. P-values were calculated using the Wilcoxon test. (C) Ferroptosis score distributions in cell types of spatial data in ICH-1h and ICH-24h. (D) Heatmap showing significantly highly activated ferroptosis in oligodendrocytes. (E) Ferroptosis score distributions in different brain regions of spatial data in ICH-1h and ICH-24h. (F, G) Annotated cryosection on the spatial transcriptome slide (left) and the MIA map of all cell types identified using scRNA-seq and spatial transcriptome-defined histological regions (right). Red indicates enrichment and blue indicates depletion.

We evaluated PCD signature scores to determine which PCD process was enriched after ICH. Using SsGSEA, we assessed five PCD processes using well-established gene sets from the GO and Kyoto Encyclopedia of Genes and Genomes databases^14^. After ICH, ferroptosis scored considerably higher than the other four PCDs (Figure 2A). Ferroptosis was observed as early as 1 hour post-ICH and peaked at 24 hours post-ICH (Figure 2B). Oligodendrocytes showed significant activation of ferroptosis at 24 hours after ICH (Figure 2C). Furthermore, a heatmap demonstrated that oligodendrocytes had more ferroptosis than other cells (Figure 2D). Spatial transcriptomics revealed that ferroptosis was first distributed highly in the hippocampus, and then in the choroid plexus at 24 hours post-ICH (Figure 2F, G).

These findings demonstrate that ferroptosis is the primary PCD mechanism following ICH; it began as early as 1 hour post-ICH, peaked at 24 hours post-ICH, and persisted for up to 7 days post-ICH. Additionally, ferroptosis gene expression was predominantly observed in oligodendrocytes and was localized to the hippocampus initially after ICH, followed by distribution in the choroid plexus.

### Oligodendrocyte ferroptosis is involved in pathological processes after ICH

**Figure 3:**
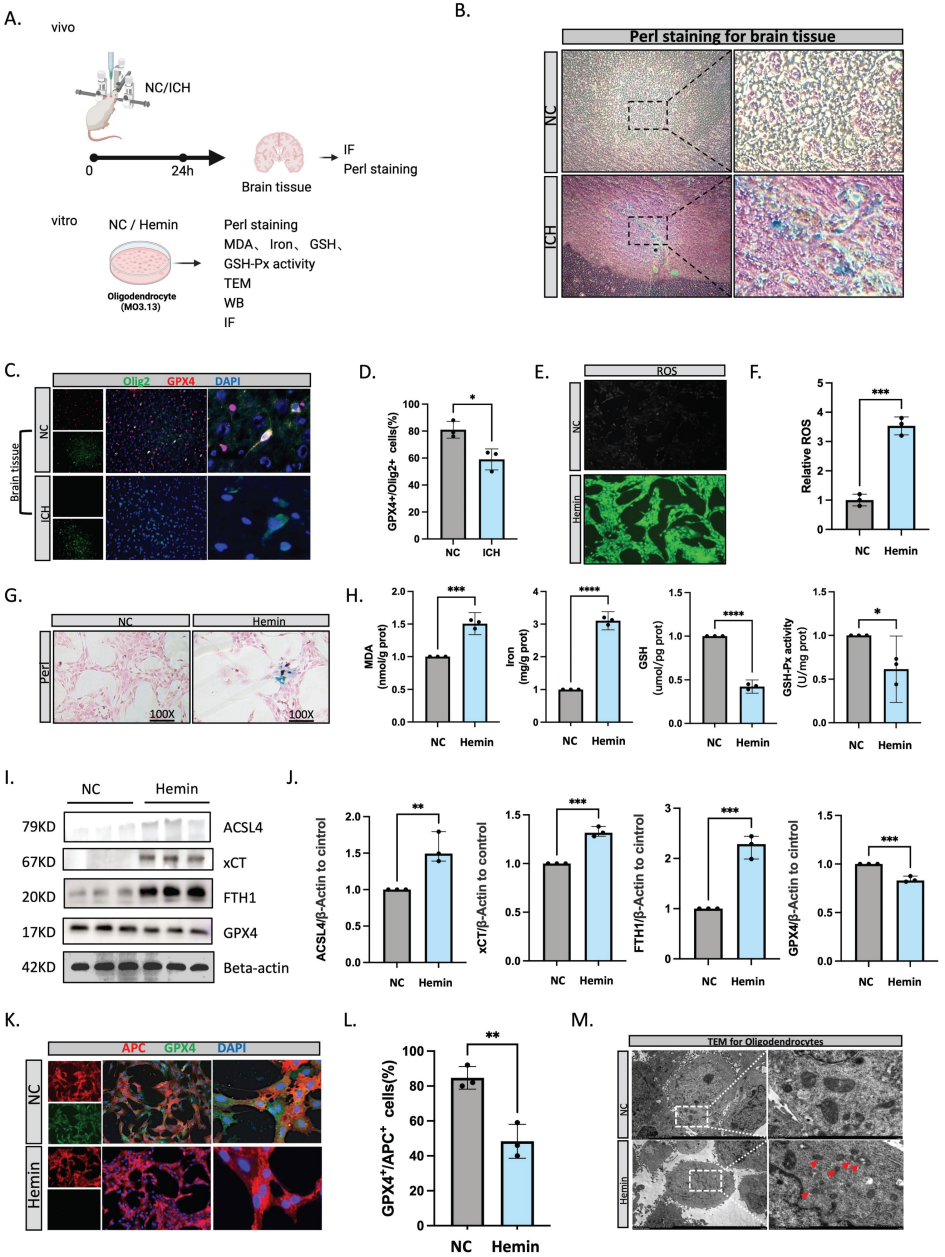
** Oligodendrocyte ferroptosis is involved in pathological processes after ICH.** (A) Flow diagram. (B) Perl staining for rat brain tissue. (C, D) Immunofluorescent staining of OLIG2/GPX4 in rat brain tissue at 24 hours after ICH (n = 3, bar = 20 μm). (E, F) ROS levels assessed using a kit for oligodendrocytes (n = 3, bar = 20 μm). (G) Perl staining for oligodendrocytes. (H) Malondialdehyde (MDA), iron, GSH, and GSH-Px activity levels were assessed using a kit (n = 3). (I, J) Western blot bands of ferroptosis-related molecules from oligodendrocytes and the densitometric quantification of ACSL4, xCT, FTH1, and GPX4 (n = 3). (K, L) Immunofluorescent staining of APC/GPX4 for oligodendrocytes (n = 3, bar = 20 μm). *P < 0.05; **P < 0.01; ***P < 0.001; ****P < 0.0001. (M) Transmission electron microscopy of oligodendrocytes showing vacuolization in mitochondria. Scale bar = 500 nm.

To further confirm the occurrence of ferroptosis in oligodendrocytes following ICH, we conducted both in vitro and in vivo experiments (Figure 2A). Using Perls staining, we quantified the accumulation of cellular iron. Notably, cells with increased iron content were abundant and were significantly expanded within the brain tissue by 24 hours post-ICH (Figure 2B). Immunofluorescent staining revealed that GPX4^+^/OLIG2^+^ (a marker of oligodendrocyte lineage) cells were decreased at 24 hours after ICH compared with normal brain tissue (Figure 3C, D). Hemin is the oxidized form of heme, and is released from hemoglobin following erythrocyte lysis. Notably, hemin has been implicated in secondary injury following ICH, and the treatment of oligodendrocytes with 100 μM of hemin can be used to simulate the onset of cerebral hemorrhage^26^. Furthermore, the accumulation of lipid peroxidation is a hallmark of ferroptosis^27^. We therefore assessed lipid ROS accumulation using C11 BODIPY 581/591 fluorescent probes. Malondialdehyde content is an end-product of lipid peroxidation, and glutathione synthesis (GSH) of antioxidants plays an indispensable role in preventing lipid peroxidation during ferroptosis^28^. In the present study, hemin increased lipid ROS and iron, but reduced GSH and GSH-Px activity (Figure 3E-H). Next, we evaluated genes that are vital for ferroptosis at the protein level, such as ACSL4 (an essential component for ferroptosis execution), xCT (a subunit of system χc-importing cystine), FTH1, and GPX4. Following hemin treatment, oligodendrocytes had higher ACSL4, xCT, and FTH1 levels and lower GPX4 levels (Figure 3I, J). Furthermore, after hemin treatment, there was less GPX4^+^/APC^+^ (myelin-producing mature oligodendrocytes) co-localization (Figure 3K, L). Transmission electron microscopy revealed that hemin-induced oligodendrocytes had smaller mitochondria, fragmented and folded mitochondrial ridges, and vacuoles (Figure 3M).

### Ferroptosis is preferred in OLs over OPCs following ICH

**Figure 4:**
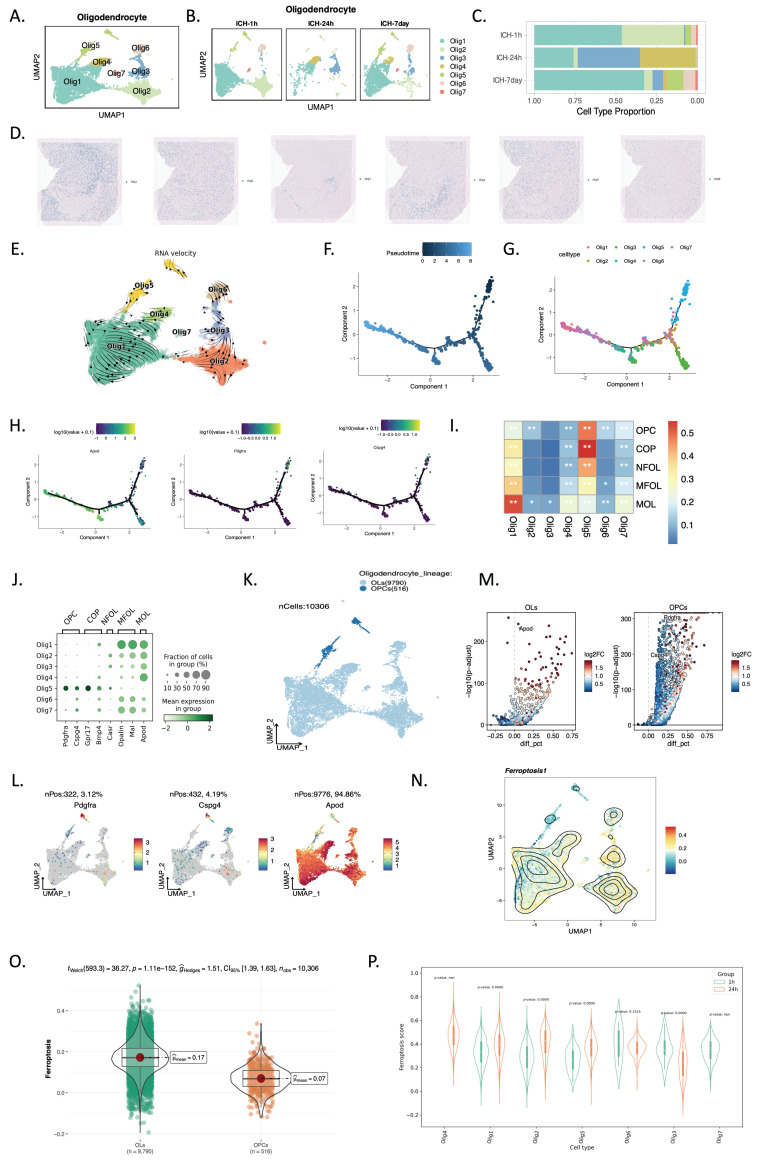
** scRNA-seq and spatial RNA-seq profiling identified two oligodendrocyte lineages of OPCs and Ols.** (A, B) UMAP visualization of 10,306 oligodendrocytes (A) across three time points (B; ICH-1h: n = 5,814, ICH-24h: n = 1,418, ICH-7d: n = 3,074). (C) Relative proportion of each cell type at each time point. Cell clusters are color coded corresponding to (A, B). (D) Oligodendrocyte subtype identification results of Slide-seq V2 cerebellum data by SpatialScope (24 hours post-ICH). (E) Grid visualization of RNA velocity for oligodendrocyte cell subtypes on UMAP embedding. (F, G) Potential trajectory of all oligodendrocyte cells identified two distinct cell fates, colored by pseudotime (F) and cluster (G). (H) Expression dynamics of APOD, PDGFRA, and CSPG4 along with the pseudotime. (I) Heatmap of Pearson correlations between oligodendrocyte types in our study and the five stages of oligodendrocyte lineage differentiation as reported by Marques et al. (J) Dot plot visualization of gene expression profiles across various oligodendrocyte cell types. The size of each dot reflects the fraction of cells within the group that expresses a particular gene. The color intensity of the dots denotes the mean expression level of the gene within that group, with the scale ranging from -2 to 2. (K) UMAP visualization of two oligodendrocyte lineages of OPCs and OLs. (L) UMAP visualization of the expression profiles of PDGFRA, CSPG4, and APOD. (M) Upregulated genes in OLs and OPCs. (N) UMAP visualization of ferroptosis signature scores. (O) Differences in ferroptosis signature scores between OLs and OPCs. (P) Ferroptosis score distributions in oligodendrocyte subtypes of spatial data from ICH-1h and ICH-24h.

After hemorrhagic stroke, oligodendrocyte (Olig) clusters were analyzed for heterogeneity to determine ferroptosis sites, which revealed seven subclusters (Figure 4A). Following this, we noted that Olig4 dominated at 24 hours after ICH. At other time points, Olig4 distribution was minimal; it was primarily concentrated at 24 hours (Figure 4B, C). In the oligodendrocyte subtype identification results from the Slide-seq V2 cerebellum data by SpatialScope (24 hours post-ICH), Olig4 was mainly distributed in the hippocampus and choroid plexus (Figure 4D). We used RNA velocity and pseudotime analysis to organize related clusters during oligodendrocyte development and identify OPCs and OLs after ICH. Olig5 was closer to the root and Olig3 and Olig4 were near the terminal point in the monocle pseudotime plot (Figure 4E-H). We then compared our data with existing scRNA-seq datasets of OL lineage cells to better define the post-stroke oligodendrocyte subgroups^18^. Remarkably, the transcriptomic profile of Olig5 was highly consistent with that of OPCs and COPs (Figure 4I), a transitional subtype between OPCs and OLs^29^. Furthermore, platelet-derived growth factor receptor α (PDGFRA) and chondroitin sulphate proteoglycan 4 (CSPG4) were strongly expressed by Olig5, whereas apolipoprotein D (APOD) was scarcely expressed (Figure 4J). We then displayed the two oligodendrocyte lineages of OPCs and OLs and their specific markers using UMAP, and displayed their upregulated genes in OLs and OPCs in heatmaps (Figure 4K-M). We subsequently assessed ferroptosis signature scores in two cell lines and found that OLs had higher scores (Figure 4N, O). Next, we analyzed ferroptosis score distributions in oligodendrocyte subtypes based on spatial data in ICH-1h and ICH-24h; Olig4 had higher scores than the other subtypes (Figure 4N, O). Together, these findings suggest spatial overlaps in the expression of ferroptosis-related and *OLIG4* genes.

### Single-cell and spatial transcriptome profiling showed LCN2^+^ microglia in the ICH-24h rat brain

**Figure 5:**
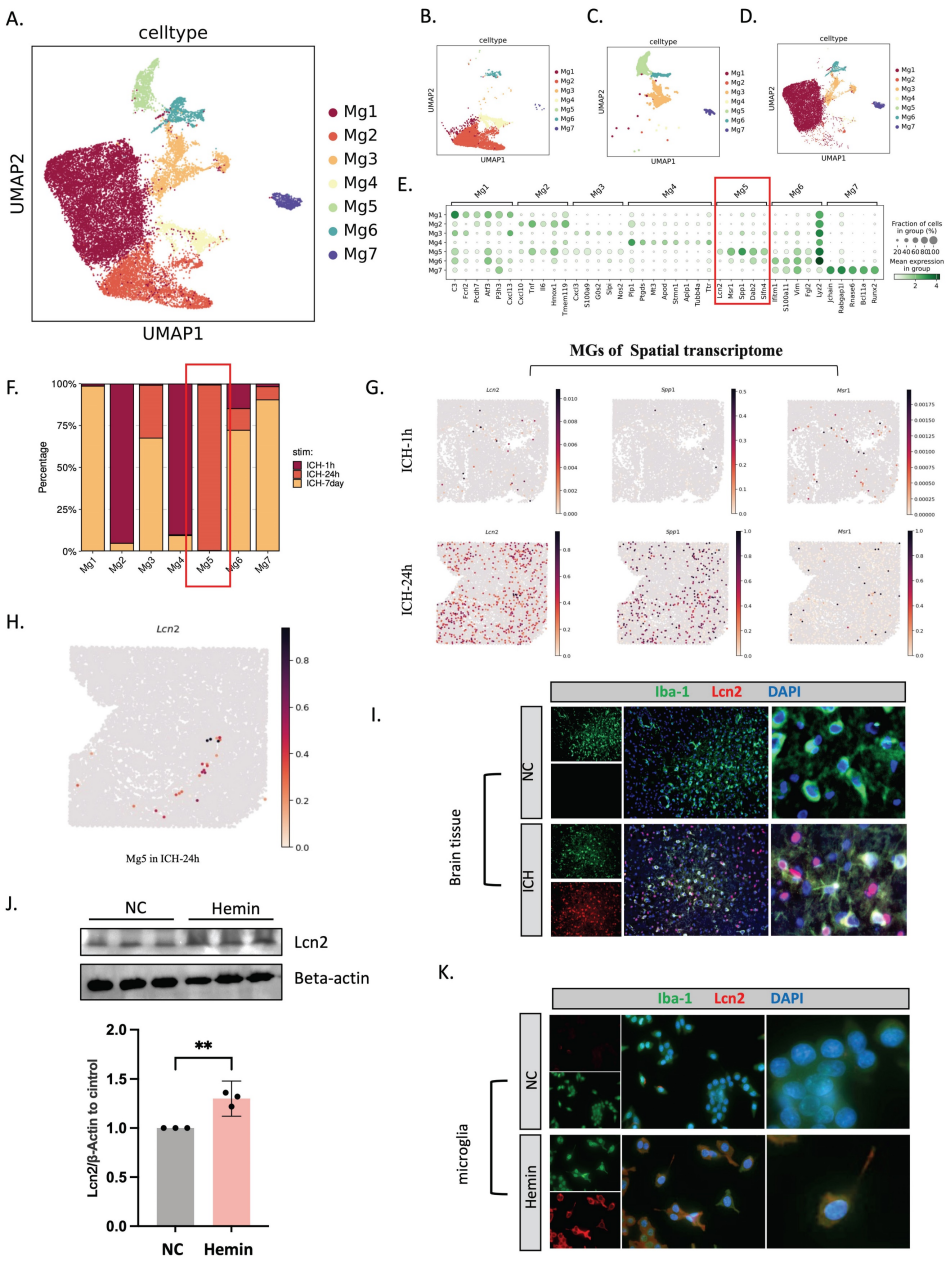
** scRNA-seq and spatial RNA-seq profiling reveals LCN2^+^ microglia in the ICH-24h rat brain.** (A-D) UMAP visualization of 18,938 microglia (A) across three time points (B-D; ICH-1h: n = 4,763, ICH-24h: n = 1,685, ICH-7d: n = 12,490). (E) Dot plot visualization of gene expression profiles across various microglial cell types. (F) Relative proportion of each cell type at each time point. (G) Visualization of LCN2, SPP1, and MSR1 expression in the microglial identification results of Slide-seq V2 cerebellum data by SpatialScope (top: 1 hour post-ICH, bottom: 24 hours post-ICH). (H) Spatial mapping of LCN2 in Mg5 of ICH-24h. (I) Immunofluorescent staining of ionized calcium-binding adapter molecule 1 (Iba-1)/LCN2 in rat brain tissue at 24 hours after ICH (n = 3, bar = 20 μm). (J) Western blot bands and densitometric quantification of LCN2 and beta-actin (n = 3 per group). (K) Immunofluorescent staining of Iba-1/LCN2 in microglia (HMC3 cells) at 24 hours after hemin treatment (n = 3, bar = 20 μm).

Seven subclusters of microglia (Mg) were grouped in an unsupervised manner from the total cells (Figure 5A, E). UMAP revealed microglial subpopulation temporal alterations and proportional shifts (Figure 5B-D, F). At 24 hours post-ICH, the Mg5 subgroup predominated, but was barely evident at 1 hour and at 7 days (Figure 5F). We noted that Mg5 highly expressed LCN2, macrophage scavenger receptor types I and II (MSR1), and sphingosine-1-phosphate phosphatase 1 (SPP1) (Figure 5E), and their expression levels were significantly higher at 24 hours than immediately after ICH (Figure 5G). Spatial mapping showed the distribution of LCN2 in Mg5 of ICH-24h (Figure 5H). Immunofluorescent staining demonstrated increased LCN2^+^ microglia in brain tissue 24 hours after ICH (Figure 5I). We then treated HMC3 cells with hemin. After hemin treatment, the microglia expressed more LCN2 in western blot and immunofluorescent assays (Figure 5J, K).

### LCN2^+^ microglia-mediated ferroptosis in oligodendrocytes after ICH

**Figure 6:**
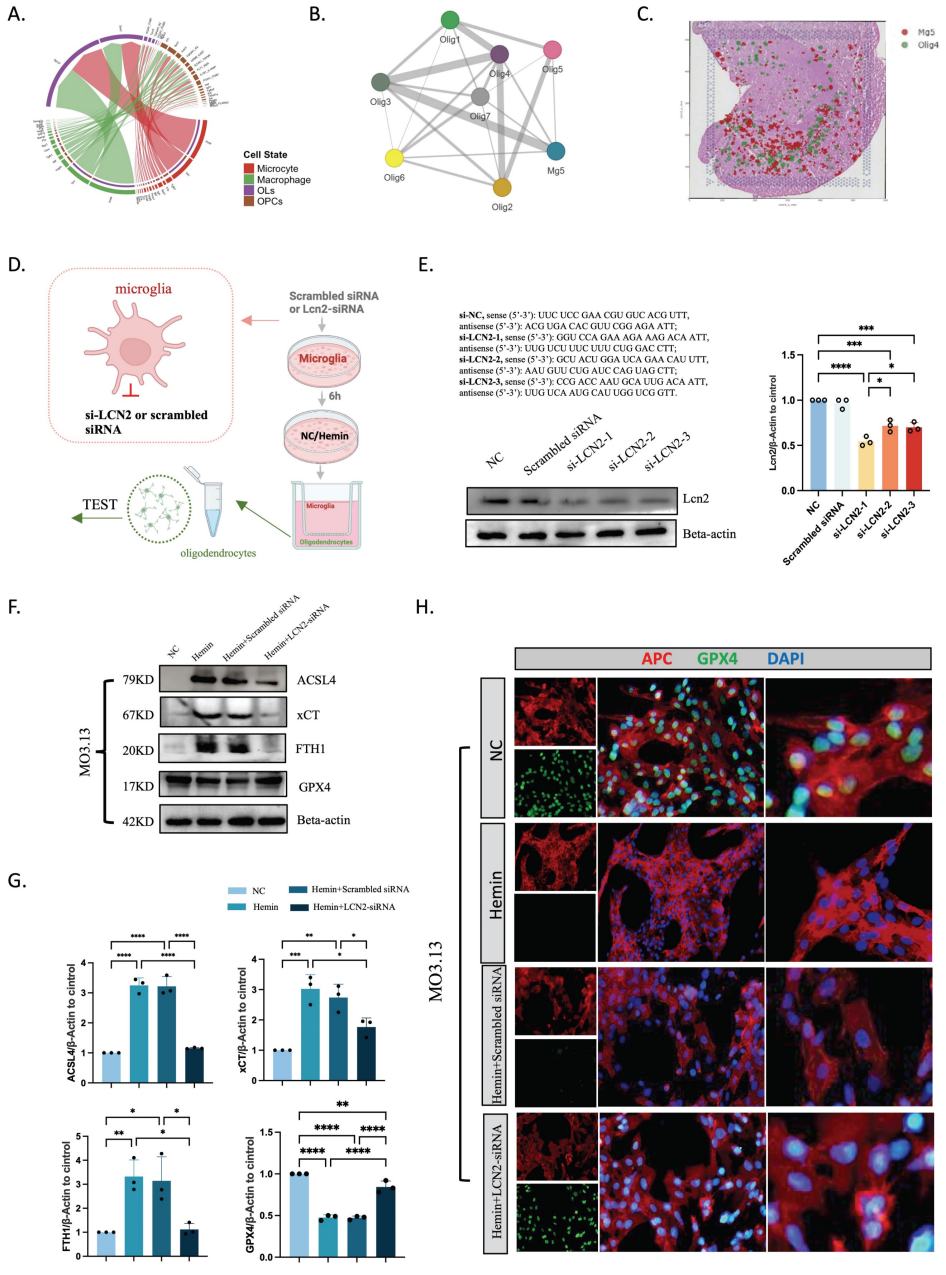
** Intercellular communication between microglia and oligodendrocytes.** (A) Intercellular communication among glial cells, as revealed by CellChat. (B) Spatial colocalization graph of Mg5 and oligodendrocyte cell types using SColoc. (C) CellTrek map of scRNA-seq data from Mg5 and Olig4. (D) Flow diagram. (E) Western blot bands and densitometric quantification of LCN2 (n = 3). *P < 0.05; **P < 0.01; ***P < 0.001; ****P < 0.0001. (F, G) Western blot bands and densitometric quantification of ACSL4, xCT, FTH1, and GPX4 (n = 3). *P < 0.05; **P < 0.01; ***P < 0.001; ****P < 0.0001. (H) Immunofluorescent staining of APC/GPX4 in oligodendrocytes at 24 hours after ICH (n = 3, bar = 20 μm).

CellChat revealed that microglia and oligodendrocytes were related (Figure 6A), and the spatial transcriptome demonstrated that the Mg5 and Olig4 subpopulations of microglia and oligodendrocytes were strongly correlated and spatially distributed (Figure 6B, C). We then created a co-culture setup to study LCN2^+^ microglia-oligodendrocyte interactions. HMC3 cells were stimulated with hemin or PBS at 24 hours after 6-8 hours of si-NC or si-LCN2 pretreatment. For 2 days, HMC3 cells were co-cultured in the top chambers and MO3.13 cells were cultured in the bottom. We subsequently collected oligodendrocytes for ferroptosis-related gene analysis (Figure 6D-H). Western blot showed that the protein levels of ACSL4, xCT, and FTH1 were lower and GPX4 was higher in oligodendrocytes in the hemin+si-LCN2 group than in the hemin+si-NC or hemin groups (Figure 6F, G). Moreover, immunofluorescent staining revealed that the number of APC^+^/GPX4^+^ cells was lower in the hemin+si-LCN2 group than in the hemin+si-NC group (Figure 6H).

### LCN2 inhibition attenuates neurological deficits

**Figure 7:**
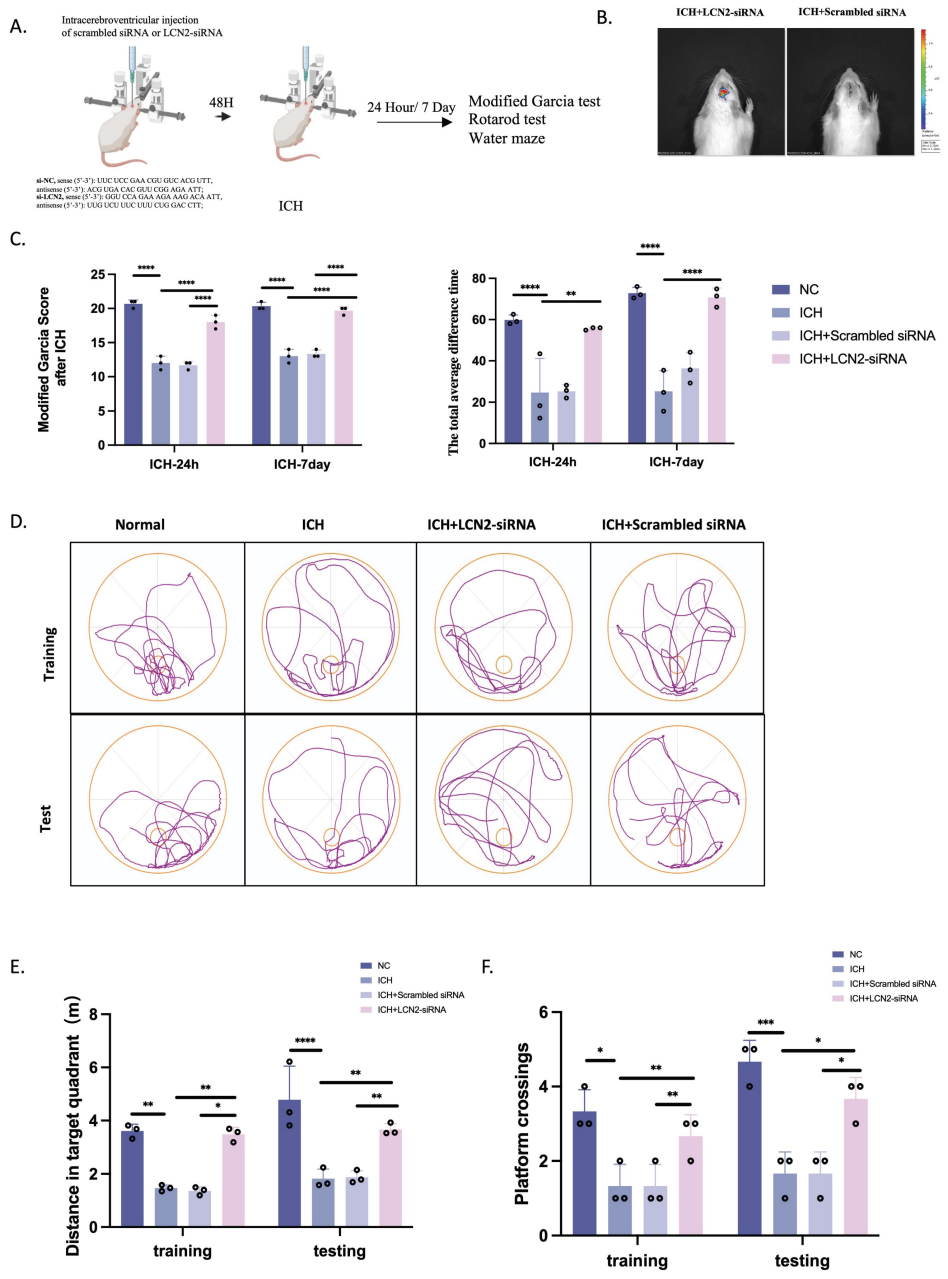
** LCN2 inhibition attenuates neurological deficits.** (A) Flow diagram. (B) Live imaging of rats. (C-F) Neurobehavioral functions were assessed using the modified Garcia test **(C, left)**, rotarod test **(C, right),** and Morris water maze **(D-F)** at 24 hours and 7 days following ICH (n = 3). *P < 0.05; **P < 0.01; ***P < 0.001; ****P < 0.0001.

We treated rats with si-LCN2 to study its function and target value (Figure 7A). In vivo imaging confirmed its translocation into brain tissue after labeling tiny interferences with green fluorescent protein-fluorescent tags (Figure 7B). We next measured the neurobehavioral functions of rats using the modified Garcia test, rotarod test, and Morris water maze at 24 hours and 7 days following ICH induction (Figure 7C-F). Neurological deficits assessed by the modified Garcia test were significantly improved at 24 hours and 7 days after ICH in the ICH+si-LCN2 group compared with the ICH group (Figure 7C). The rotarod test revealed that sensorimotor performance deficits were notably improved in the ICH+si-LCN2 group at both 24 hours and 7 days post-ICH; the total average time spent on the rotarod apparatus was the highest compared with ICH rats (Figure 7C). Moreover, the Morris water maze demonstrated that rats in the ICH+si-LCN2 group had better spatial learning and memory impairments compared with the ICH group (Figure 7D-F).

### LCN2^+^ microglia-mediated ferroptosis in oligodendrocytes via CSF1/CSF1R after ICH

**Figure 8:**
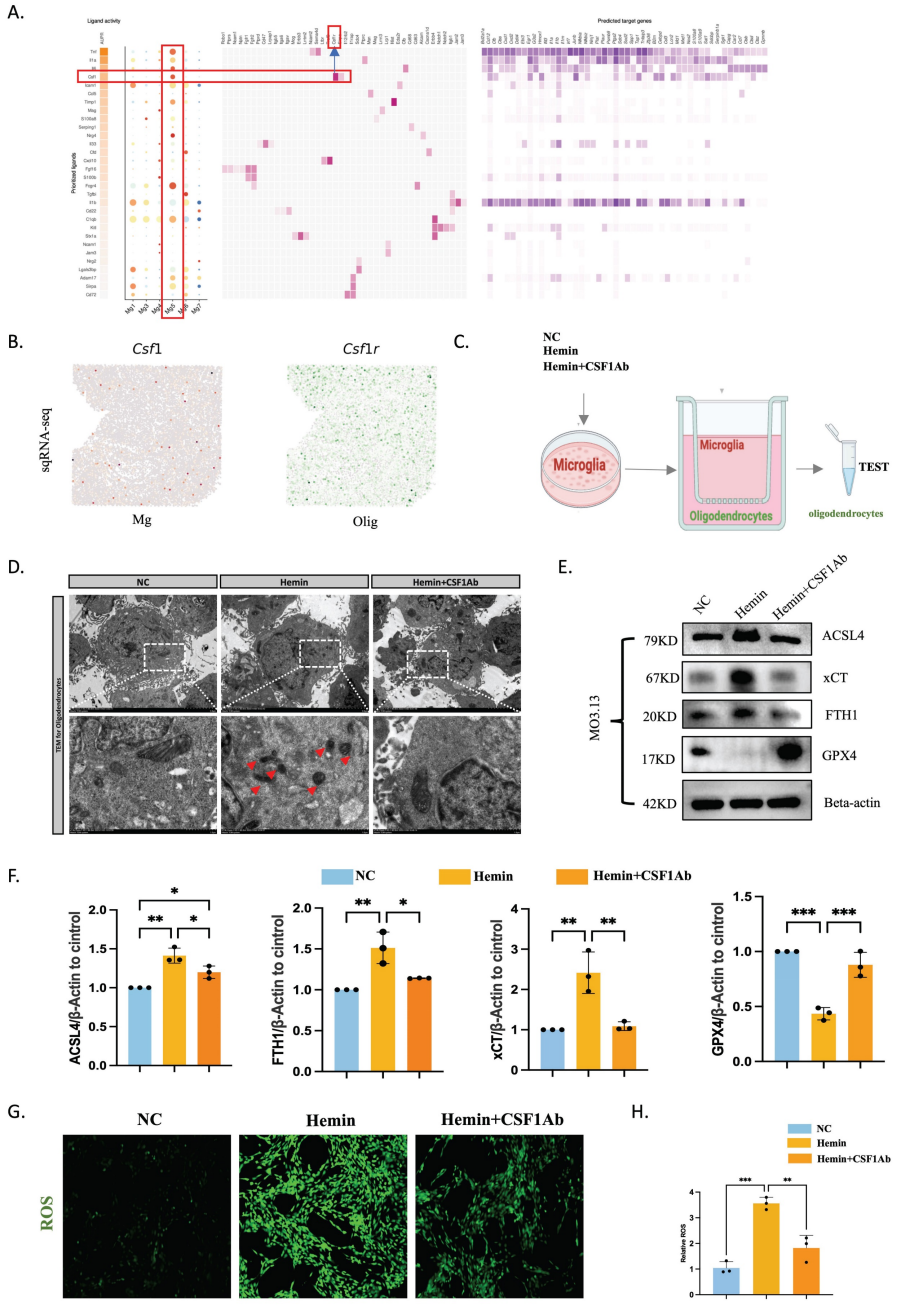
** LCN2^+^ microglia induce oligodendrocyte ferroptosis via the CSF1/CSF1R pathway.** (A) Top-ranked ligands that were inferred to regulate oligodendrocytes by LCN2^+^ microglia (Mg5) according to NicheNet. Dot plots showing the expression percentages (dot size) and intensities (dot intensity) of top-ranked ligands in each microglial subtype. Ligand-receptor pairs showing interactions between LCN2^+^ microglia and oligodendrocytes were ordered by ligand activity. Heatmap showing the regulatory potential of the top 20 ranked ligands and downstream target genes in oligodendrocytes. (B) Visualization of CSF1 in microglia and CSF1R in oligodendrocytes in the identification results of Slide-seq V2 cerebellum data by SpatialScope (24 hours post-ICH). (C) Flow diagram of the co-culture system. (D) Transmission electron microscopy of oligodendrocytes showing vacuolization in mitochondria. Scale bar = 500 nm. (E, F) Western blot bands and densitometric quantification of ACSL4, xCT, FTH1, and GPX4 (n = 3). *P < 0.05; **P < 0.01; ***P < 0.001; ****P < 0.0001. (G, H) ROS levels were assessed using a kit for oligodendrocytes (n = 3, bar = 20 μm).

Single-cell transcriptomics revealed the strongest correlation between microglia Mg5 and oligodendrocytes according to NicheNet, and ligand-receptor pairs showed interactions between Mg5 and oligodendrocytes ordered by CSF1/CSF1R (Figure 8A). Slide-seq V2 cerebellum data by SpatialScope identified CSF1 in microglia and CSF1R in oligodendrocytes (24 hours post-ICH) (Figure 8B). This revelation came from our finding that the Mg5 microglial subset, characterized by heightened *LCN2* gene expression, acts as the principal reservoir of CSF1. After secretion, this factor engages with CSF1R that adorn the surface of oligodendrocytes, thereby choreographing a sophisticated intercellular dialogue. To validate this idea, we deployed neutralizing antibodies targeting CSF1 (CSF1Ab) to modulate microglial function within an ICH model. Our experimental framework incorporated two control cohorts, thus ensuring the integrity and reliability of our findings. Microglia were treated with NC, hemin, or hemin+CSF1Ab, and were then co-cultured with oligodendrocytes. The ensuing effect on ferroptosis-related markers within oligodendrocytes was explored using western blotting, immunofluorescence, transmission electron microscopy, and ROS techniques (Figure 8C-H). Transmission electron microscopy demonstrated that mitochondria were smaller, mitochondrial ridges were broken and folded, and vacuoles were formed within them in oligodendrocytes in the hemin group compared with the hemin+CSF1Ab group (Figure 8D). Western blotting revealed that the oligodendrocyte protein levels of ACSL4, xCT, and FTH1 were lower and GPX4 was higher in the hemin+CSF1Ab group than in the hemin group (Figure 8E, F). Furthermore, the ROS level was lower in the hemin+CSF1Ab group than in the hemin group (Figure 8G, H). Together, these findings indicate that LCN2^+^ microglia-mediated ferroptosis occurs in oligodendrocytes via CSF1/CSF1R after ICH.

## Discussion

Although many studies have reported that different PCDs contribute to poor ICH outcomes and that PCDs can occur throughout the different ICH pathophysiological processes, few studies have explored which PCD is most critical after ICH progression. To discern the predominant mode of cell death and its timing as well as the cell types and brain regions that are most affected following brain hemorrhage, we performed single-cell and spatial transcriptome sequencing of rat brain tissue at 1 hour, 24 hours, and 7 days post-ICH. In our investigation, ferroptosis was the most abundant PCD process in ICH, and oligodendrocytes scored the highest. This process emerged at 1 hour post-ICH, peaked at 24 hours post-ICH, and lasted until 7 days post-ICH. Ferroptosis was preferably distributed in the hippocampus and choroid plexus. Additionally, a microglial subset with strong LCN2 expression promoted ferroptosis in oligodendrocytes after ICH. In terms of the underlying mechanisms, we revealed that LCN2^+^ microglia caused oligodendrocyte ferroptosis via the CSF1/CSF1R pathway, and that suppressing this pathway protected against neurological impairments.

Ferroptosis is a PCD process that is characterized by intracellular iron accumulation and lipid ROS build-up^9^. It arises when iron-mediated free radicals induce lipid peroxidation in the absence of adequate antioxidant repair mechanisms. Following ICH, hemoglobin is released from degraded red blood cells, and microglia and macrophages engulf this hemoglobin around the hematoma, releasing ferrous/ferric iron^30^. This iron accumulation within neurons generates free radicals and ROS, thus damaging cell membranes, proteins, and DNA, ultimately leading to cell death^31^. Although numerous studies have suggested the important role of ferroptosis in ICH pathogenesis, the mechanisms underlying iron dysregulation in this context remain unclear. In the present study, we calculated PCD scores across various post-ICH stages using single-cell and spatial transcriptomics. The ferroptosis scores notably surpassed other PCD scores, peaking at 24 hours and persisting for up to 7 days post-ICH. Furthermore, ferroptosis-related genes were predominantly expressed in the hippocampus and choroid plexus after ICH. Oligodendrocytes exhibited the highest ferroptosis activity in ICH pathogenesis, followed by astrocytes. Oligodendrocytes participate in myelin formation, accurate wrapping, and repair during development and in various diseases^6^. Given its relatively high iron content, low levels of antioxidant agents (such as glutathione peroxidase), and reduced glutathione reductase activity compared with other glial cells, the oligodendroglial lineage is particularly susceptible to oxidative stress^32^. Wu et al^33^. reported that interleukin (IL)-10 alleviates hemin-induced lipid ROS accumulation and ferroptosis in OPCs via the IL-10/STAT3/DLK-1/ACC axis. To confirm that ferroptosis occurs in oligodendrocytes post-ICH, we conducted both in vitro and in vivo experiments, as outlined in Figure 3.

Previous research has demonstrated that OPCs originate in the subventricular zone and migrate to the corpus callosum, striatum, and fimbria fornix to differentiate into mature OLs in adult mice^34^. In the present study, we conducted a detailed analysis of differentiation trajectories from OPCs to mature OLs (Figure 4) and found that OLs exhibited higher ferroptosis scores compared with OPCs. A spatial analysis of oligodendrocyte subtypes using Slide-seq V2 cerebellum data by SpatialScope (24 hours post-ICH) revealed that Olig4 was mainly distributed in the hippocampus and choroid plexus, which was consistent with our observation that ferroptosis-related genes were predominantly expressed in these regions following ICH. Together, these findings suggest that ferroptosis in oligodendrocytes may play a crucial role in causing neurological deficits post-ICH, and highlight the potential of oligodendrocyte ferroptosis regulation as a novel therapeutic strategy for ICH.

Microglia monitor the surroundings of the brain and initiate immune responses^35^. Early investigations have demonstrated that activated microglia destroy oligodendrocytes^36^. During the present heterogeneity analysis of microglia, we categorized microglia into seven distinct subpopulations. We observed that Mg5 exhibited a significant increase in expression 24 hours post-hemorrhagic stroke, whereas this population was scarcely expressed at 1 hour or 7 days post-hemorrhage. However, Mg5 accounted for over 80% of the total microglial population at the 24-hour mark, emerging as the predominant subpopulation at that time point. Mg5 highly expressed LCN2 (also known as neutrophil gelatinase-associated lipocalin), which is a member of the lipocalin family^37^. LCN2 is also an oxidative stress marker and is reportedly upregulated in response to oxidative stress^38^. Our scRNA-seq and spatial RNA-seq data revealed the strongest correlation between microglia Mg5 and oligodendrocytes. Previous studies have highlighted the importance of LCN2 in iron regulation during hemorrhagic stroke^37^. Furthermore, Shin et al. demonstrated that LCN2 deficiency reduces hippocampal iron overload and oxidative stress, suggesting that it may be involved in iron-related oxidative stress and neuroinflammation^39^. Our findings indicate that LCN2 downregulation reduces iron levels and ferroptosis-related gene expression (ACSL4, xCT, and FTH1) in oligodendrocytes, but increases GPX4 expression. Moreover, LCN2 silencing in rats improved ICH-related neurobehaviors, suggesting that microglial LCN2 inhibition may suppress oligodendrocyte ferroptosis and alleviate neurological deficits post-ICH. Our ligand-receptor pair results revealed that Mg5 interacted with Olig4 via CSF1/CSF1R. CSF1 is produced by various normal cells across different tissue types. Its role in inflammatory diseases has been reported in studies involving CSF1-deficient MRL-Faslpr mice and Csf1op/Csf1op mice with unilateral ureteral obstruction, in which reductions in lupus-like disease and renal inflammation were observed^40^. Further studies have indicated that treatment with an anti-CSF1 antibody significantly attenuates dextran sodium sulfate-induced colitis, thus highlighting the involvement of CSF1 in mucosal inflammation^41^. Moreover, our findings are derived from the observation that the Mg5 microglial subset, characterized by robust *LCN2* gene expression, serves as the primary source of CSF1. Upon secretion, this factor binds to CSF1R on the surface of oligodendrocytes, thus initiating a complex intercellular communication. To validate this concept, we used CSF1Ab in an ICH model to modulate microglial activity^42^, ^43^. The experimental design included two control groups to ensure the validity and reliability of our results. Microglia were treated with NC, hemin, or hemin+CSF1Ab before being co-cultured with oligodendrocytes. Ferroptosis in oligodendrocytes was decreased following CSF1Ab treatment, suggesting that LCN2^+^ microglia induce ferroptosis in oligodendrocytes through the CSF1/CSF1R pathway following ICH, and indicating the potential neuroprotective properties of microglial LCN2 silencing for attenuating oligodendrocyte ferroptosis and subsequent neurological deficits.

Our study, although insightful, is not without its limitations. First, ethical constraints prevented the acquisition of human brain tissue samples from ICH patients, which hinders the direct confirmation of our findings in a clinical context. This gap underscores the need for the further substantiation of our results. Second, although the ICH model that we used is supported by existing literature, it may not fully replicate the complexity of ICH that is observed in human patients. Further exploration of more accurate modeling methods is therefore warranted to enhance preclinical fidelity.

## Conclusions

Our findings indicate that ferroptosis is the predominant form of PCD in ICH, and particularly affects oligodendrocytes. Ferroptosis initiates within 1 hour post-ICH, peaks at 24 hours, and persists up to 7 days post-ICH, occurring predominantly in the hippocampus and choroid plexus. We also identified a subset of microglia that expresses high levels of LCN2 as promoters of ferroptosis in oligodendrocytes post-ICH. Mechanistically, we demonstrated that these LCN2^+^ microglia induce oligodendrocyte ferroptosis via the CSF1/CSF1R signaling pathway; inhibiting this pathway may mitigate neurological damage.

## Figures and Tables

**Figure 9:**
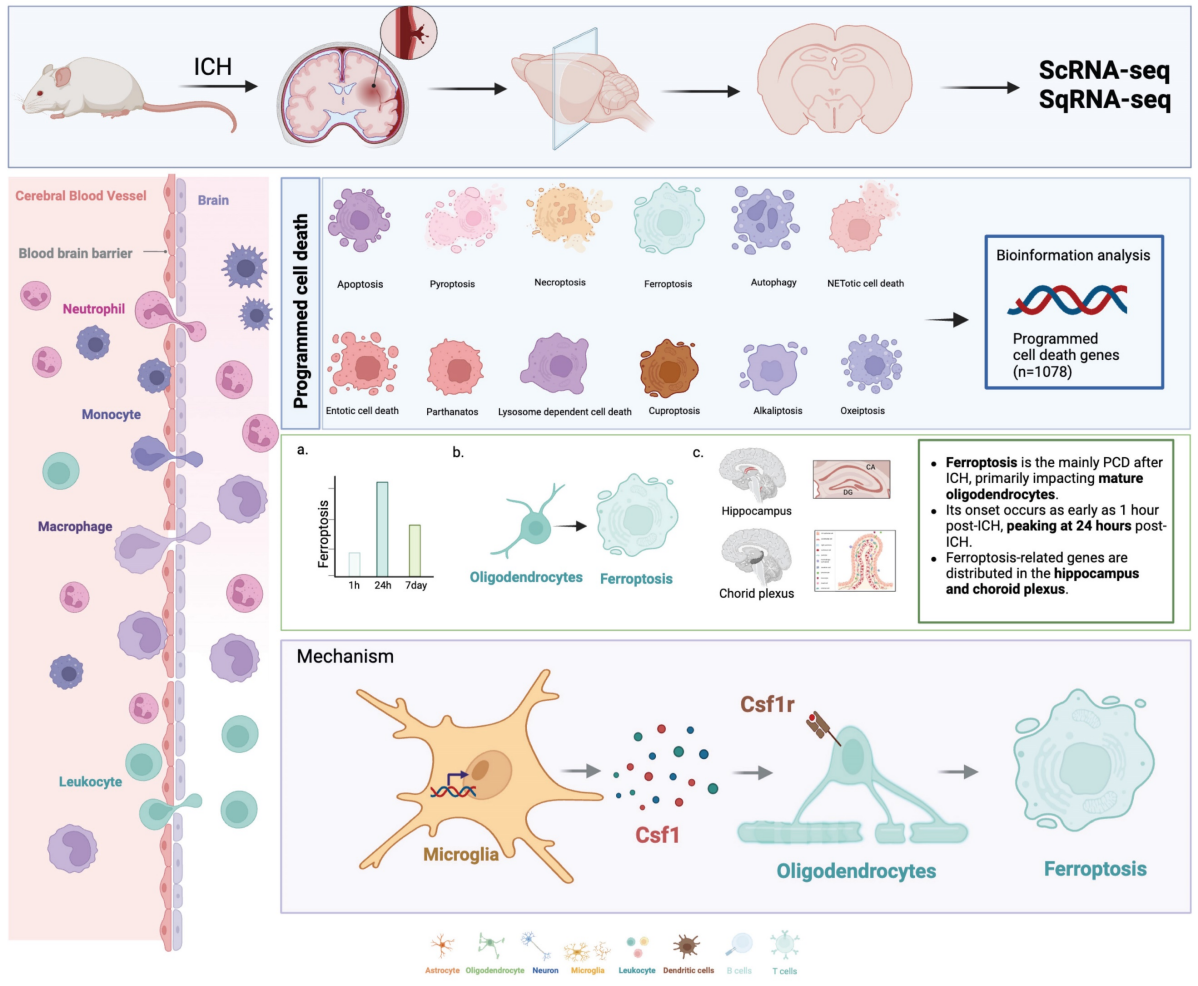
** Ferroptosis-Mediated Neurodegeneration in Rat Brain Post-Hemorrhagic Stroke: Spatiotemporal Gene Expression and Cellular Interactions.** Single-cell and spatial transcriptomic techniques were used to investigate PCD-related gene expression trends in rat brain tissue following hemorrhagic stroke. Ferroptosis was the primary form of PCD after ICH, and predominantly affected mature oligodendrocytes. Its onset was observed as early as 1 hour post-ICH, and peaked at 24 hours post-ICH. Ferroptosis-related genes were predominantly distributed in the hippocampus and choroid plexus. We delineated a specific interaction between LCN2^+^ microglia and oligodendrocytes that was mediated by the CSF1/CSF1R pathway; this culminated in ferroptosis induction in oligodendrocytes and subsequent neurological deficits.

## Abbreviations

ICH: Intracerebral hemorrhage
PCD: Programmed cell death
OPCs: oligodendrocyte precursor cells
SBI: secondary brain injury
WM: white matter
ROS: reactive oxygen species
MDA: Malondialdehyde
GSH: Glutathione synthesis
FTH1: ferritin heavy chain
TEM: Transmission electron microscopy
ACSL4: acyl-CoA synthetase long-chain family member 4
MOLs: Mature oligodendrocytes
CNS: central nervous system
CC: corpus callosum
Lcn2: Lipocalin-2
NGAL: neutrophil gelatinase-associated lipocalin
